# Comprehensive Gene Expression Analysis in NMIBC Using RNA-seq Reveals New Therapy Strategies

**DOI:** 10.3389/fonc.2019.00523

**Published:** 2019-06-25

**Authors:** Xiaoliang Chen, Fuquan Jiang, Chunshu Jia, Ming Liu, Yonghao Nan, Licheng Qu, Qingkuo Kong, Fangfang Hou, Wenshan Luo, Wanli Na, Xuefei Jin, Jiufeng Tan

**Affiliations:** ^1^Department of Urology, China-Japan Union Hospital of Jilin University, Changchun, China; ^2^Centre for Reproductive Medicine, Centre for Prenatal Diagnosis, First Hospital of Jilin University, Changchun, China; ^3^Central People's Hospital of Siping City, Siping, China; ^4^Department of Urology, the Frist Affiliated Hospital of Zhengzhou University, Zhengzhou, China

**Keywords:** NMIBC, modularization, WGCNA, PPI, biomarker

## Abstract

Non-muscle invasive bladder cancer (NMIBC) patients often have fewer treatment options, and suffer the progression of disease due to mechanisms that are not clear, as well as due to its diversity. This study was designed to explore the molecular mechanism of bladder cancer through an RNA-seq. In addition to conventional analyses, we also simplified the network through modularization using the WGCNA algorithm, with the help of the topological overlapping matrix and hierarchical cluster tree, which are based on the PPI network of STRING. Furthermore, the hub genes were confirmed through survival analyses in the independent cohorts (*n* = 431). Among them, 15 genes were significantly associated with poor prognosis. Finally, we validated the results at mRNA and protein level using qRT-PCR, IHC and western blotting. Taken together, our research is important for the prediction, as well as the prospective clinical development of drug targets and biomarkers.

## Introduction

Bladder cancer is a prevalent disease among the world, which is mainly attributed to smoking (1). Men are more likely to be affected than women, and morbidity increases with age (2). Bladder cancer grows through two distinct pathways: non-muscle invasive type and muscle invasive type. The majority of bladder patients are diagnosed through macroscopic hematuria, and diagnosis is confirmed after surgical resection, which is the primary stage of treatment (3). Although the 5 years survival rate as a result of current therapies is more than 80% for non-muscle invasive bladder cancer (NMIBC) patients, while a recurrence rate of nearly 70% results in patients being under lifelong surveillance and makes NMIBC the most expensive cancer from diagnosis to death (4, 5). Therefore, new therapeutic strategies are necessary to overcome these challenges.

Recent studies of bladder cancer based on gene expression profiles have gradually elucidated the molecular mechanism of the disease (6). Except for the transcriptional features have been discovered through earlier traditional microarrays, many molecular characteristics have been identified through integrative analyses (7, 8). Indeed, a lot of putative biomarkers and drug targets, including FGFR3, VEGF, CEBPA, and CCNE1, have been identified in different investigations (9–12). However, none of them have probed the regulation of their expression and of their associated genes, which could provide an extra perspective into the molecular mechanisms of disease progression.

In this study, we performed an RNA-seq on non-muscle bladder cancer patients who were subjected to surgical resection. After data processing, the reads were aligned with hg38 using STAR, and DESeq was used to filter the differentially expressed genes (DEGs). Then, we used these DEGs to execute functional annotation, including GO and KEGG enrichment analyses, which were further validated using GSEA. In order to simplify the network and identify functional clusters, modularization analysis was established through WGCNA and integrated with the PPI network from STRING, in order to model the dynamics of proteome changes. Afterwards, survival analysis was used to assess the clinical outcomes of hub genes, which are located as connections in each module. Finally, the results were confirmed through experiments, and it is hoped that they may be used as a reference for gene therapy for bladder cancer.

## Results

### Identification of Differentially Expressed Genes (DEGs) and Functional Variation

**Figure 1:**
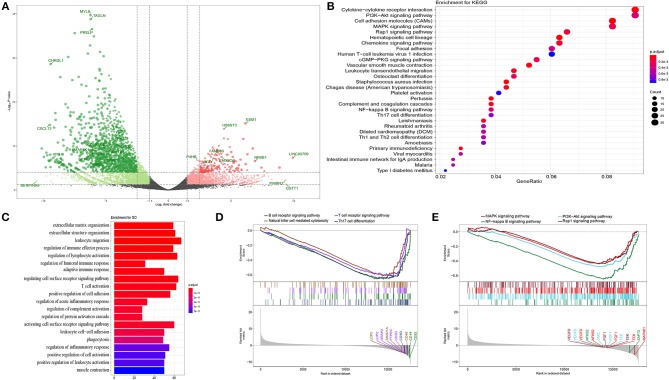
Functional annotation. **(A)** Volcano plot of DEGs in bladder cancer compared with adjacent tissue. *P*-value were counted by the Wald test. Candidate hub genes were shown with different colors. **(B)** Bubble plot of top 30 enrichment of KEGG pathways. *P*-values were shown by different color, the size of bubble indicate the gene count of each pathway. **(C)** GO enrichments were arranged by top 20 significantly p values. **(D,E)** GSEA of whole transcription from RNA-seq, *p*-value was made by Kolmogorov-Smirnov test.

DEGs were screened out using the DESeq package depending on read counts at transcription level, which were identified using an absolute log_2_FC value >1 and adjusted *p*-value of <10^5^ as the statistical conditions for filtering. We obtained 885 DEGs, including 54 significantly upregulated genes and 831 significantly downregulated genes between the bladder cancer tissue and adjacent tissue. A volcano plot was used to visualize the results, in which hub genes and significantly changed genes were indicated (Figure 1A).

In order to further analyze the DEGs, we explored functional variation between the two groups using the clusterProfiler package. 430 GO terms were identified with an adjusted *p*-value of <0.01. The GOSemSim package was used to remove similar terms by keeping only one representative term, which resulted in 142 unique GO terms (13). The top 20 are shown in Figure 1C. Even though the extracellular matrix and organization structure were the most statistically changed functions, many terms involved in the immune response and activation, such as the regulation of lymphocyte activation, regulation of humoral immune response, adaptive immune response, and T cell activation, were also found. KEGG analysis also revealed many DEGs related to the downstream pathways of immune activation (Figure 1B), such as the PI3K-Akt signaling pathway, MAPK signaling pathway, and NF-kappa B signaling pathway. Most DEGs were downregulated in these processes. Recently, an unsupervised clustering by cytogenetic analysis divided NMIBC into two subtypes, no cytogenetic changes subtype (genomic subtype1, GS1) and another subtype with loss of 9q in chromosome (GS2). GS2 often appear in high grade tumors, and loss some regulators of AKT/PI3K/mTOR pathway. This may be why the dysfunction of AKT/PI3K pathway in NMIBC (14). In order to further verify the relationship between phenotype and functionally changed genes, we performed GSEA on the whole genome at transcription level. The transcripts of bladder cancer were remarkably associated with downregulated genes related to T and B cell receptor signaling pathways and their downstream pathways, which is in accordance with GO and KEGG enrichment analysis (Figures 1D,E).

### Integrative Network Analysis Reveals New Functional Modules

**Figure 2:**
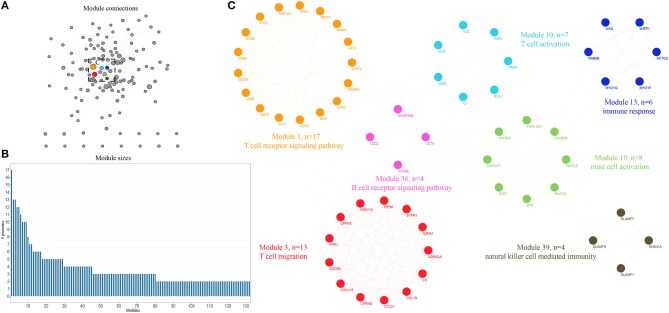
Expression profiling of whole proteome reveals co-expression clusters and functional modules in bladder cancer. **(A)** Allocation of 132 modules. Each node represents the individual module and their interactions by the module size. Edges connect modules that share PPIs. Boxed modules are further enlarged in **(C)**. **(B)** Distribution of modules size, modules were identified by superimposition of proteins in bladder cancer onto the PPI network. The numbers of members from each module are exhibited in the figure. **(C)** Seven interconnected modules of immune reaction system derived from bladder cancer, with showing the protein names and representative functional terms.

An integrative analysis method was used to model the dynamics of proteome changes upon cancer progression, as described previously (15). In brief, we applied WGCNA to all DEGs in order to cluster the correlative proteins that had similar molecular functions or biological processes (16). Later, these proteins were superimposed onto the PPI network in order to identify functional modules. As a result, we identified 132 modules, with the number of proteins ranging from 17 to 2 (Figure 2B), and 117 out of the 132 modules were highly interconnected through their members (Figure 2A). Each module was annotated by known functional terms or signaling pathways. For instance, the modules were remarkably enriched in the immune reaction system including the T cell-mediated immune response (module 1, 13, 26, and 34), B cell-mediated immune response (module 11, 28, 34, and 38), mast cell activation (Module 6, 10, and 51) and natural killer cell mediated immunity (Module 39, 41, 47, and 75). Furthermore, some of the modules involved in cell invasion and migration processes also contributed to the progression of tumorigenesis, as commonly known, through mechanisms such as extracellular matrix organization (Module 4, 72) and the integrin-mediated signaling pathway (Module 32, 51). In summary, progression of bladder cancer is through the rebalanced regulation and extensive reprogramming of mutually connected functional modules.

### Survival Analysis of Hub Genes

**Figure 3:**
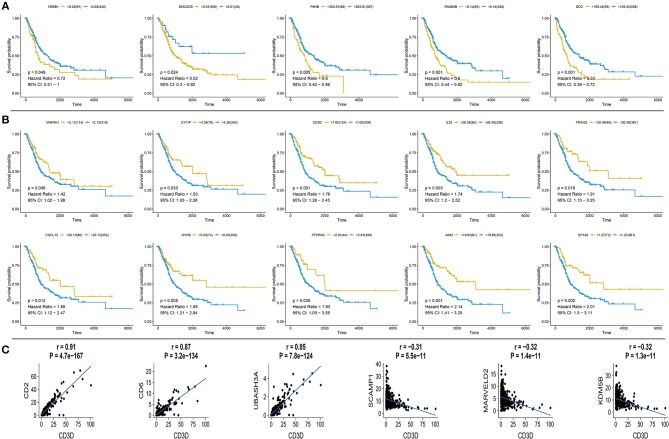
Survival curve for hub genes of 433 TCGA bladder cancer samples and analysis of Pearson's correlation of CD3D **(A)** Five up-regulated hub genes with poor prognosis in bladder cancer, *p*-value, Hazard ration, and 95% CI are shown. **(B)** Ten down-regulated hub genes with poor prognosis in bladder cancer, p value, Hazard ration and 95% CI are shown. **(C)** Pearson's correlation of CD3D with top 3 positive and negative genes in bladder cancer from TCGA database, relevant gene expression versus change upon CD3D, *p*-value and correlation are shown.

Based on the expression profile and clinical data of 431 bladder cancer samples from TCGA database, the clinical outcomes of hub genes that are indicated in Figures 1, 2 were evaluated through survival analysis. 15 out of the 62 hub genes were significantly associated with poor prognosis, and were either positively or negatively correlated with a higher risk and were either upregulated or downregulated with bladder cancer (Figure 3). Among the hub genes, CD3D was the core factor of the network, which was involved in the T cell receptor signaling pathway and was connected to T cell and mast cell activation. We computed the Pearson correlation of CD3D using 26,483 transcripts of 431 bladder cancer patients. CD2, CD6, and UBASH3A were the most positively correlated genes, while CD3D, and SCAMP1, MARVELD2, and KDM5B were the most negatively correlated. These genes might also be involved in the regulation of bladder cancer progression, and might also be candidate biomarkers or drug targets for the disease.

### Initial Validation of Transcriptome Results Using qRT-PCR and IHC

**Figure 4:**
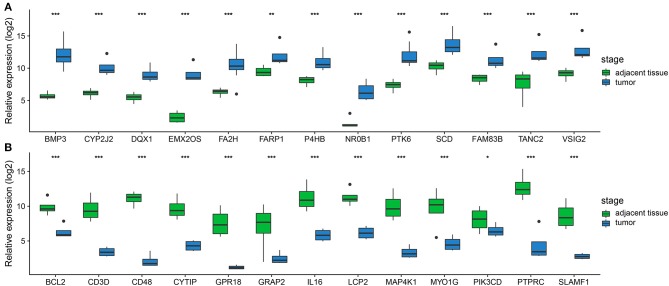
Validation of DEGs by qRT-PCR. **(A,B)** Boxplots indicate the medians and dispersions of 24 bladder cancer and their adjacent tissue samples. *P*-values are counted by student's *t*-test, *p < 0.05, **p < 0.01, ***p < 0.001.

**Figure 5:**
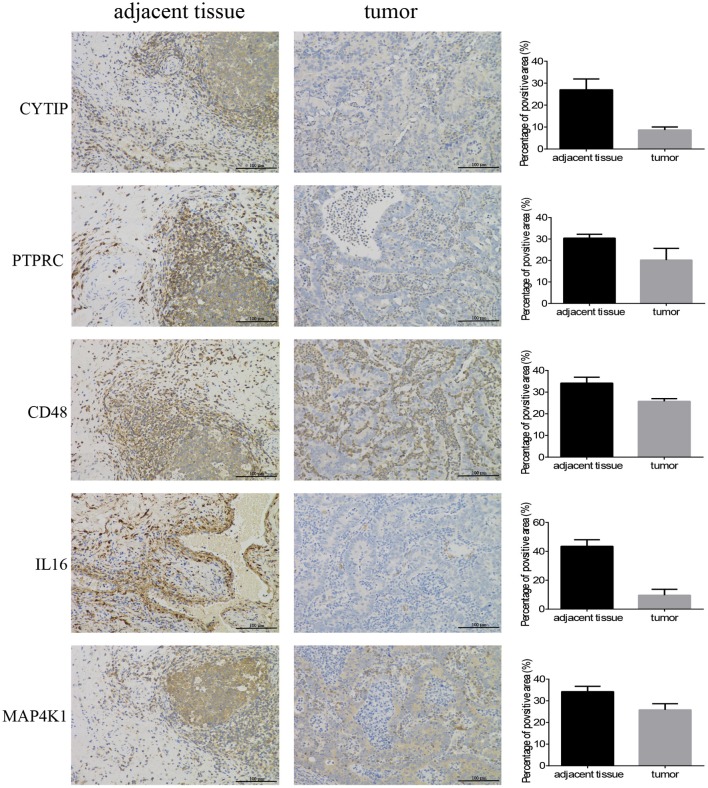
Immunohistochemistry. Five hub genes expression in 12 pairs of bladder cancer and adjacent tissues (magnification 200×).

In order to confirm the DEGs found through the experiment, total RNA of 24 paired tumor tissues were isolated for qRT-PCR validation. Twenty six target DEGs were selected as shown in Figure 4. Moreover, IHC was also performed to further validate the five target genes of patients who underwent surgical resection (Figure 5). In brief, the DEGs were successfully validated and showed good correspondence with the analysis of transcriptome, indicating that the RNA-seq results were precise and reliable.

### Signaling Pathway Validation Using Western Blotting

**Figure 6:**
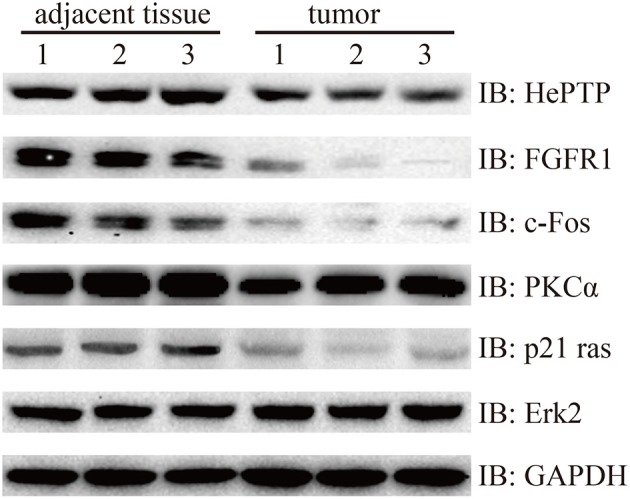
Western blotting detection of MAPK signaling pathway. Lysates from three pairs of bladder cancer and adjacent tissues were subjected to western blotting with antibody to HePTP, FGFR1, c-Fos, PKCα, p21 ras, and Erk2. GAPDH is a reference gene.

Finally, we wanted to confirm the signaling pathways at protein level. MAPKs are evolutionarily conserved kinases, ubiquitously expressed and regulate a wide range of biological processes, such as cell growth, differentiation and death (17, 18). In cancer, the MAPK signaling pathway can play a double role by either maintaining cell survival or impelling cell death, through different mechanisms (19). In this study, we found that Fibroblast growth factor receptor 1 (FGFR1), which is amplified in lung and breast cancer, was downregulated in bladder cancer samples compared with that of the controls (20, 21). FGFR1 genes are fused to TACC1 through interstitial deletions, which were also downregulated in our results (log_2_FC = −0.91). The other three genes of the MAKP signaling pathway, PKCα, p21 Ras, and c-Fos, followed the same trend as that of FGFR1. More strikingly, protein phosphatase HePTP, which is a negative regulatory factor, also performed a similar action (Figure 6).

## Discussion

It is well-known that bladder cancer is the 11th most malignant tumor worldwide, and 70% of patients present with NMIBC. However, the exact biological functional variation during the progression of bladder cancer is still obscure. In order to provide deeper insights into the molecular mechanism involved in this process, we performed an RNA-seq on three paired bladder cancer patients who underwent surgical resection at China-Japan Union Hospital of Jilin University, and made a comprehensive analysis of the results, together with data from TCGA database. We identified core DEGs, significant biological processes, pathways, and validated our results using qRT-PCR, IHC and western blotting. In general, our work revealed an interlaced network presented by central modules that are involved in bladder cancer development, in which hub genes may play an indispensable role.

We sought for the expression patterns of transcripts and functional variations between bladder cancer tissue and adjacent tissues using RNA-seq, which produced a massive amount of data. In order to extract useful information from the large amount of data to explain the molecular mechanism of bladder cancer, in our study, we focused on two concepts. First, the DEGs were annotated by GO and KEGG pathway analyses, and the results involved functions related with immunity, cell adhesion and cancer. Furthermore, GSEA provided a good method of validating the functional annotations of the whole genome at transcription level rather than the DEGs. We also deciphered the complex network through modularization using WGCNA superimposed onto the PPI database of STRING. Each module was facilitated through the hierarchical cluster tree and topological overlapping matrix, which echoed the annotated functions of GO and KEGG. Overall, the complicated network was simplified by modularization into modules, which made it easier for it to be learned by hub genes that were the connections among the modules. Second, the bladder cancer dataset obtained from TCGA was used to evaluate the clinical significance of the hub genes. Fifteen hub genes, including five upregulated and 10 downregulated, were associated with overall survival of patients, which indicates poor prognosis of bladder cancer. Among the hub genes, CD3D attracted our attention due to its location on the most important module. Pearson correlation was used to find the co-expression of CD3D and the expression pattern was assessed. Finally, partial hub genes were validated using qRT-PCR and IHC on specimens from the bladder cancer patients.

Along with the development and application of NGS technologies, a large number of sequencing data has been accumulated. However, we should be conscious of an analytical system that is so sophisticated that it is above our initial cognition. Fortunately, modern methodologies have provided us with a good way of simplifying complex networks, which include thousands of proteins that can be disassembled into several independent and correlated modules, and the hub genes of each module can be probed in detail. The active application of public databases promotes the elucidation of gene functions. As mentioned above, our study clearly presents the significant biological modules, pathways and hub genes involved in the progression of bladder cancer. However, some core genes might be shut out if they fail to be positioned in the modules, or have not been filtered out as DEGs, which may play an important role even though their expression does not greatly change during cancer progression. Therefore, we may have missed these genes in our analysis. Taken together, we systematically analyzed the molecular mechanism of functional variation in bladder cancer through biological modules and hub genes, which were confirmed using qRT-PCR, IHC, and western blotting. The revelation that they are involved in tumor progression could be used to design new strategies to treat aggressive carcinoma. For example, the downregulation of CD3D in bladder cancer samples and the T-cell receptors that are essential for the activation of T cell signaling, indicate a new therapeutic approach for bladder cancer. In addition, similarly, other hub genes may also prove to be useful drug targets and prognostic markers in gene therapies.

## Methods

### Patients and Samples

All specimens were obtained from bladder cancer patients between April 2016 and December 2017 at China-Japan Union Hospital of Jilin University (Changchun, China), with the approval of the Ethics Committee. The samples were surgically resected followed by being treated with liquid nitrogen, and were then stored at −80°C. According to routine procedure, all samples were assessed using HE staining and diagnosis was made by three independent pathologists.

### RNA-Seq and Data Processing

Total RNA of the three bladder cancer tissues and their paired adjacent tissues were isolated using Trizol reagent (Sangon Biotech, Shanghai, China), as described in other studies. The cDNA libraries were constructed using a custom protocol, which were sequenced using the Illumina Hiseq 2500 sequencer (Sangon Biotech, Shanghai, China). Raw data were uploaded to Sequence Read Archive (SRA) (PRJNA525544).

The adaptor sequences of raw reads were removed using cutadapt (22), then the clean reads were aligned to the human genome (hg38) using STAR (23). Prior to the next analysis, the R package, DESeq (24), was used to remove bad counts and filter the differential expression according to the conditions of an absolute log_2_FC value of >1 and an adjusted *p*-value of <10^5^.

### Functional Analysis

The DEGs were used to perform GO and KEGG analyses using the clusterProfiler package (25), and an adjusted *p*-value of <0.01 was considered as a significant event. Moreover, in order to deeply analyze functional variations between the bladder cancer tissue and their adjacent tissue, GSEA was utilized to discover the molecular mechanism of the whole genome at transcription level, rather than the DEGs.

### Network Analysis

All DEGs were used for the co-expression analysis using the WGCNA package (26) and were superimposed onto the PPI database of STRING (27). The co-expression analysis clusters were delineated using the dynamic tree cut package, with the minimum height for each module set to 0.2 (28). The overall trend of each module was based upon the eigengene, and the members of each module were collected through Pearson correlation from among DEGs and their interactors. Moreover, a topological overlapping matrix was also utilized to filter the PPI network (29). In the end, individual modules were annotated using clusterProfiler (25) and were visualized in Cytoscape (30).

### Survival Analysis

The survival analysis was used to reveal the clinical outcomes of the hub genes in cancer prognosis. The expression profiles and clinical data of 431 bladder cancer patients were obtained from TCGA database using TCGAbiolinks (31). The 431 samples were split into a high expression group and a low expression group, according to the hub genes, using the survminer package for the best separation. A *p*-value of <0.05 was considered statistically significant and is shown in the results.

### Quantitative Real-Time PCR (qRT-PCR)

qRT-PCR was used to verify the results of RNA-seq. The total RNA of 24 paired bladder tumors and their adjacent tissues were extracted using TRIzol. The genes of interest were then quantified through qRT-PCR using a One-Step qPCR Kit (Invitrogen, USA) and executed with a CFX ConnectTM Real-Time System (BIO-RAD, USA), according to the manufacturer's instructions. The results were analyzed through the 2^−ΔΔCT^ method (32), with GAPDH as a reference gene.

### Immunohistochemistry (IHC)

The specimens from the bladder patients who underwent surgical resection were cut to 4 μm thick sections, were then formalin-fixed and paraffin-embedded for IHC, as described previously (33). The primary antibodies used are as follows: CD48 (No.133506, Abcam), MAP4K1 (No.33910, Abcam), IL16 (No.184161, Abcam), CYTIP (No.154847, Abcam), and PTPRC (No.40763, Abcam). Image Pro Plus 6.0 (Media Cybernetics, Bethesda, MD, United States) was employed to measure the positive area of hub genes for quantitative analysis.

### Western Blotting

The tissue samples were stored at −80°C for 16 h and lysed with Tissue Extraction Reagent I (Invitrogen, USA) supplemented with protease and phosphatase inhibitors. The BCA assay kit (Thermo Scientific, USA) was then used to measure protein concentration. In brief, the lysate proteins were separated using SDS-PAGE, followed by being transferred into PVDF membranes (Invitrogen, USA), then subjected to the general process of western blotting, according to the instructions of the manufacturers of the antibodies, purchased from CST and Santa Cruz: PKCα (#59754, CST), FGFR1 (#9740, CST), c-Fos (#2250, CST), HePTP (sc-271245, Santa Cruz), p21 Ras (#3965, CST), Erk2 (#9108, CST) and GAPDH (#5174, CST).

### Statistical Analysis

All experiments were performed in triplicate, at least. For the analysis between two groups, the student's *t*-test was leveraged for comparison between tumor tissue and its adjacent tissue. Data are presented as mean ± SDs, except when indicated otherwise. A *p*-value of <0.05 is considered to be statistically significant.

## Data Availability

The datasets generated for this study can be found in Sequence Read Archive (SRA), PRJNA525544.

## Ethics Statement

This study was carried out in accordance with the recommendations of CIOMS. The protocol was approved by the institutional review boards of the China-Japan Union Hospital of Jilin University. All subjects gave written informed consent in accordance with the Declaration of Helsinki.

## Author Contributions

XJ and JT conceived and designed the study. XC, FJ, CJ, and ML collected analyzed the data, XC and FJ wrote the manuscript. YN, LQ, QK, FH,WL, and WN collected the samples and revised the manuscript. All authors read and approved the manuscript.

### Conflict of Interest Statement

The authors declare that the research was conducted in the absence of any commercial or financial relationships that could be construed as a potential conflict of interest.
